# Health-Related Workplace Absenteeism Among Full-Time Workers — United States, 2017–18 Influenza Season

**DOI:** 10.15585/mmwr.mm6826a1

**Published:** 2019-07-05

**Authors:** Matthew R. Groenewold, Sherry L. Burrer, Faruque Ahmed, Amra Uzicanin, Sara E. Luckhaupt

**Affiliations:** ^1^Division of Field Studies and Engineering, National Institute for Occupational Safety and Health, CDC; ^2^Emergency Preparedness and Response Office, National Institute for Occupational Safety and Health, CDC; ^3^Division of Global Migration and Quarantine, National Center for Emerging and Zoonotic Infectious Diseases, CDC.

During an influenza pandemic and during seasonal epidemics, more persons have symptomatic illness without seeking medical care than seek treatment at doctor’s offices, clinics, and hospitals (*1*). Consequently, surveillance based on mortality, health care encounters, and laboratory data does not reflect the full extent of influenza morbidity. CDC uses a mathematical model to estimate the total number of influenza illnesses in the United States (*1*). In addition, syndromic methods for monitoring illness outside health care settings, such as tracking absenteeism trends in schools and workplaces, are important adjuncts to conventional disease reporting (*2*). Every month, CDC’s National Institute for Occupational Safety and Health (NIOSH) monitors the prevalence of health-related workplace absenteeism among full-time workers in the United States using data from the Current Population Survey (CPS) (*3*). This report describes the results of workplace absenteeism surveillance analyses conducted during the high-severity 2017–18 influenza season (October 2017–September 2018) (*4*). Absenteeism increased sharply in November, peaked in January and, at its peak, was significantly higher than the average during the previous five seasons. Persons especially affected included male workers, workers aged 45–64 years, workers living in U.S. Department of Health and Human Services (HHS) Region 6* and Region 9,^†^ and those working in management, business, and financial; installation, maintenance, and repair; and production and related occupations. Public health authorities and employers might consider results from relevant absenteeism surveillance analyses when developing prevention messages and in pandemic preparedness planning. The most effective ways to prevent influenza transmission in the workplace include vaccination and nonpharmaceutical interventions, such as staying home when sick, covering coughs and sneezes, washing hands frequently, and routinely cleaning frequently touched surfaces (*5*).

CPS is a monthly national survey of approximately 60,000 households conducted by the U.S. Census Bureau for the Bureau of Labor Statistics. The survey collects information on employment, demographics, and other characteristics of the civilian, noninstitutionalized population aged ≥16 years; CPS is the nation’s primary source of labor force statistics. Data on all sample household members are collected from a single respondent by trained interviewers using a standardized questionnaire during in-person or telephone interviews (*3*). During July 2016–June 2018, the response rates ranged from 84% to 88%.^§^

A full-time worker is defined as an employed person who reports usually working ≥35 hours per week. Health-related workplace absenteeism is defined as working <35 hours during the reference week because of the worker’s own illness, injury, or other medical issue. Because CPS questions refer to 1 week of each month, absenteeism during the other weeks is not measured. These 1-week measures are intended to be representative of all weeks of the month during which they occur.

Each month, NIOSH updates an influenza season–based time series of the prevalence of health-related workplace absenteeism among full-time workers with the previous month’s estimate (i.e., with a 1-month lag). Point estimates and 95% confidence intervals (CIs) are calculated and compared with an epidemic threshold defined as the 95% upper confidence limit of a baseline established using data from the previous five seasons, aggregated by month (*6*). Estimates with lower 95% confidence limits that exceed the epidemic threshold are considered significantly elevated. Estimates by sex, age group, geographic region (HHS Regions^¶^), and specific occupational group** are also calculated.

Using these data, health-related workplace absenteeism prevalence during the high-severity 2017–18 influenza season (October 2017–September 2018) was analyzed. All analyses were weighted using the CPS composite weight, and estimates of all standard errors were adjusted to account for the complex design of the CPS sample. Analyses were performed using SAS software (version 9.4; SAS Institute).

The prevalence of health-related workplace absenteeism among full-time workers was 1.7% (95% CI = 1.6%–1.8%) in October 2017, increased sharply beginning in November, peaked in January 2018 at 3.0% (95% CI = 2.8%–3.2%), and declined steadily thereafter to a low of 1.4% (95% CI = 1.3%–1.5%) in July before gradually increasing again in August and September (Table). The January absenteeism peak significantly exceeded the epidemic threshold (Figure 1). Absenteeism remained elevated in February, but not significantly. Peak absenteeism in the 2017–18 influenza season exceeded that of any of the five previous seasons except the 2012–13 season (Figure 2).

**TABLE:** Monthly prevalence of health-related workplace absenteeism* among full-time workers^†^ during the 2017–2018 influenza season, by sex, age group, U.S. Department of Health and Human Services (HHS) region^§^ and occupational group — Current Population Survey, United States, October 2017–September 2018

Characteristic	Weighted % (95% CI)											
	2107			2018								
	Oct	Nov	Dec	Jan	Feb	Mar	Apr	May	Jun	Jul	Aug	Sep
**Overall**	**1.7 (1.6–1.8)**	**1.8 (1.6–1.9)**	**2.3 (2.1–2.4)**	**3.0 (2.8–3.2)^¶^**	**2.7 (2.5–2.9)**	**2.2 (2.0–2.3)**	**2.0 (1.8–2.1)**	**1.8 (1.6–1.9)**	**1.7 (1.6–1.8)**	**1.4 (1.3–1.5)**	**1.6 (1.4–1.8)**	**1.9 (1.7–2.0)**
**Sex**												
Male	1.4 (1.3–1.5)	1.4 (1.2–1.5)	1.9 (1.7–2.1)	2.6 (2.4–2.9)^¶^	2.3 (2.1–2.4)^¶^	1.9 (1.7–2.1)	1.7 (1.5–1.8)	1.3 (1.2–1.5)	1.5 (1.3–1.6)	1.2 (1.0–1.4)	1.5 (1.3–1.7)	1.6 (1.3–1.8)
Female	2.1 (1.9–2.4)	2.3 (2.0–2.5)	2.8 (2.5–3.0)	3.6 (3.3–3.8)	3.2 (2.9–3.5)	2.5 (2.4–2.7)	2.3 (2.1–2.5)	2.4 (2.1–2.7)	2.1 (1.9–2.3)	1.7 (1.5–1.9)	1.8 (1.6–2.0)	2.2 (2.0–2.4)
**Age group (yrs)**												
16–24	1.8 (1.3–2.3)	1.7 (1.2–2.3)	2.0 (1.4–2.6)	3.2 (2.4–4.1)	2.4 (1.7–3.0)	1.6 (0.9–2.2)	1.7 (1.1–2.3)	2.2 (1.7–2.8)	1.5 (1.1–1.8)	1.4 (1.0–1.7)	1.1 (0.8–1.5)	2.0 (1.5–2.4)
25–44	1.5 (1.4–1.6)	1.6 (1.4–1.7)	2.0 (1.8–2.2)	2.5 (2.3–2.7)	2.4 (2.2–2.6)	2.0 (1.8–2.1)	1.6 (1.4–1.8)	1.5 (1.3–1.7)	1.5 (1.3–1.7)	1.2 (1.0–1.4)	1.5 (1.2–1.7)	1.7 (1.5–1.9)
45–64	1.8 (1.6–2.0)	1.9 (1.7–2.0)	2.6 (2.3–2.8)	3.4 (3.1–3.7)^¶^	3.0 (2.8–3.3)^¶^	2.4 (2.2–2.7)	2.2 (2.0–2.4)	1.8 (1.6–2.0)	1.9 (1.8–2.1)	1.5 (1.3–1.7)	1.7 (1.5–2.0)	1.9 (1.7–2.1)
≥65	3.0 (2.3–3.6)	2.6 (1.8–3.4)	3.1 (2.2–4.1)	4.6 (3.8–5.4)	3.4 (2.5–4.3)	3.2 (2.3–4.1)	4.2 (3.3–5.0)	3.2 (2.3–4.0)	2.8 (1.5–4.0)	2.6 (1.9–3.2)	2.7 (2.0–3.3)	2.7 (1.9–3.4)
**HHS region^§^**												
Region 1	1.5 (1.1–1.8)	1.7 (1.2–2.2)	2.1 (1.6–2.5)	3.0 (2.5–3.6)	2.4 (1.7–3.2)	1.5 (1.3–1.7)	2.2 (1.9–2.5)	1.5 (0.9–2.1)	1.9 (1.6–2.2)	1.7 (1.2–2.2)	1.8 (1.3–2.2)	2.0 (1.6–2.4)
Region 2	1.4 (1.1–1.7)	1.3 (0.8–1.8)	1.9 (1.6–2.1)	2.2 (1.6–2.8)	2.0 (1.6–2.5)	2.1 (1.5–2.7)	1.6 (0.8–2.4)	1.4 (1.0–1.7)	1.3 (1.2–1.4)	1.0 (0.7–1.3)	1.3 (0.2–2.5)	1.0 (0.7–1.3)
Region 3	1.5 (1.3–1.8)	1.5 (1.1–1.9)	2.6 (1.8–3.4)	2.8 (2.0–3.5)	3.2 (2.6–3.8)	2.1 (1.6–2.6)	2.4 (2.0–2.7)	1.9 (1.4–2.3)	1.9 (1.5–2.2)	1.6 (1.2–2.1)	1.3 (1.2–1.5)	2.1 (1.4–2.8)
Region 4	1.7 (1.4–2.0)	1.6 (1.4–1.8)	2.0 (1.6–2.4)	2.7 (2.3–3.1)	2.3 (1.9–2.7)	1.9 (1.8–2.0)	1.7 (1.6–1.9)	1.6 (1.4–1.8)	1.6 (1.4–1.8)	1.2 (0.9–1.5)	1.6 (1.3–1.9)	1.5 (1.2–1.9)
Region 5	1.8 (1.6–2.1)	2.1 (1.9–2.2)	2.2 (1.6–2.7)	3.2 (2.5–3.8)	3.0 (2.4–3.5)	2.3 (1.8–2.8)	2.2 (1.7–2.7)	1.8 (1.6–2.0)	1.9 (1.6–2.1)	1.3 (1.0–1.6)	1.6 (1.1–2.2)	2.1 (1.8–2.4)
Region 6	1.7 (1.6–1.8)	1.8 (1.5–2.1)	2.1 (1.8–2.3)	3.3 (3.1–3.6)^¶^	2.7 (2.4–2.9)^¶^	2.1 (1.6–2.5)	1.8 (1.5–2.1)	1.8 (1.0–2.6)	1.8 (1.4–2.2)	1.3 (0.8–1.7)	1.6 (1.3–1.9)	1.9 (1.6–2.1)
Region 7	2.2 (1.6–2.7)	2.3 (1.3–3.2)	2.3 (2.1–2.5)	2.7 (2.3–3.2)	3.0 (2.6–3.4)	2.5 (1.5–3.5)	2.4 (1.7–3.2)	1.8 (1.5–2.1)	2.2 (1.6–2.8)	1.9 (1.5–2.2)	1.6 (1.1–2.0)	1.6 (0.9–2.3)
Region 8	1.6 (0.9–2.4)	1.4 (1.2–1.6)	2.0 (1.2–2.8)	2.7 (2.4–3.0)	3.2 (1.8–4.6)	1.9 (1.5–2.3)	1.9 (1.7–2.1)	1.6 (1.2–2.1)	1.3 (1.2–1.5)	1.6 (1.3–1.9)	1.5 (0.8–2.2)	1.6 (0.9–2.3)
Region 9	1.7 (1.3–2.0)	1.9 (1.3–2.4)	2.7 (2.6–2.8)^¶^	3.5 (2.9–4.1)	2.6 (2.1–3.2)	2.7 (2.5–2.8)^¶^	1.7 (1.5–1.9)	2.1 (1.5–2.6)	1.6 (1.5–1.8)	1.6 (1.2–1.9)	1.8 (1.6–2.0)	2.2 (1.6–2.8)
Region 10	2.4 (2.1–2.6)	1.7 (1.4–2.0)	3.4 (2.0–4.7)	4.0 (3.1–4.8)	2.7 (2.4–3.1)	2.8 (2.2–3.4)	2.1 (1.5–2.6)	2.4 (1.9–2.8)	1.8 (0.7–2.9)	1.9 (1.3–2.5)	1.9 (1.4–2.5)	2.3 (2.1–2.5)
**Occupational group**												
Management, business and financial	1.2 (1.0–1.4)	1.3 (1.0–1.6)	1.7 (1.4–2.1)	2.6 (2.4–2.9)^¶^	2.1 (1.8–2.3)	1.7 (1.3–2.2)	1.6 (1.3–1.9)	1.4 (1.1–1.6)	1.2 (0.9–1.4)	1.0 (0.7–1.2)	1.1 (0.9–1.3)	1.2 (0.9–1.4)
Professional and related	1.8 (1.5–2.1)	1.6 (1.4–1.8)	2.0 (1.8–2.2)	2.8 (2.6–3.1)	2.6 (2.3–3.0)	1.8 (1.5–2.1)	1.8 (1.6–2.1)	1.6 (1.3–1.8)	1.4 (1.1–1.7)	1.2 (1.0–1.5)	1.4 (1.1–1.6)	1.6 (1.3–1.9)
Service	2.2 (1.9–2.6)	2.3 (1.8–2.7)	3.1 (2.6–3.5)	3.4 (2.8–4.0)	2.9 (2.5–3.3)	2.7 (2.2–3.2)	2.3 (1.9–2.7)	2.0 (1.7–2.4)	2.1 (1.8–2.3)	1.7 (1.4–2.0)	2.0 (1.6–2.4)	2.4 (2.0–2.8)
Sales and related	1.5 (1.1–1.9)	1.7 (1.3–2.1)	1.9 (1.4–2.4)	2.7 (2.3–3.1)	2.0 (1.5–2.4)	1.8 (1.3–2.2)	1.5 (1.1–1.8)	1.7 (1.2–2.1)	1.6 (1.1–2.1)	1.3 (1.0–1.5)	1.4 (0.9–1.8)	1.5 (1.1–1.9)
Office and administrative support	1.9 (1.5–2.3)	2.0 (1.5–2.4)	2.5 (2.1–3.0)	3.2 (2.6–3.8)	2.5 (2.1–2.9)	2.7 (2.1–3.3)	2.5 (2.1–3.0)	2.5 (2.0–3.0)	2.4 (2.0–2.8)	1.8 (1.5–2.2)	2.0 (1.6–2.4)	2.6 (2.0–3.1)
Farming, fishing and forestry	2.1 (0.7–3.4)	1.2 (0.2–2.3)	3.3 (1.4–5.2)	3.7 (1.2–6.2)	4.1 (2.4–5.7)	2.3 (0.9–3.7)	3.1 (1.1–5.2)	2.5 (0.0–6.2)	2.0 (0.0–4.2)	1.4 (0.3–2.5)	0.6 (0.0–1.4)	1.7 (0.0–3.6)
Construction and extraction	1.2 (0.8–1.5)	1.5 (1.1–1.8)	2.4 (1.8–3.0)	3.4 (2.8–3.9)	3.3 (2.5–4.0)	2.8 (2.1–3.4)	1.5 (1.0–2.1)	1.8 (1.1–2.5)	1.7 (1.1–2.3)	1.7 (1.0–2.4)	2.0 (1.4–2.5)	2.6 (1.8–3.4)
Installation, maintenance and repair	2.0 (1.2–2.7)	2.6 (1.7–3.4)	2.2 (1.5–2.9)	4.3 (3.3–5.2)^¶^	2.3 (1.4–3.2)	2.4 (1.4–3.4)	2.0 (1.3–2.6)	1.8 (1.1–2.4)	1.6 (1.1–2.1)	0.9 (0.4–1.3)	1.2 (0.7–1.7)	1.7 (1.0–2.3)
Production	1.9 (1.4–2.5)	2.1 (1.5–2.6)	2.4 (1.6–3.1)	3.2 (2.4–4.0)	4.0 (3.2–4.8)^¶^	2.6 (2.0–3.2)	2.1 (1.5–2.8)	2.0 (1.4–2.6)	2.2 (1.7–2.7)	1.9 (1.3–2.5)	2.1 (1.5–2.7)	2.1 (1.4–2.8)
Transportation and material moving	1.7 (1.3–2.2)	1.7 (1.0–2.5)	2.7 (2.1–3.3)	3.1 (2.5–3.7)	3.6 (2.9–4.3)	2.3 (1.7–3.0)	2.3 (1.7–2.9)	1.8 (1.2–2.2)	2.2 (1.7–2.8)	1.5 (1.1–2.0)	2.0 (1.4–2.6)	1.9 (1.3–2.4)

**Abbreviation:** CI = confidence interval.

* Defined as working <35 hours during the reference week because of illness, injury, or other medical issue.

^†^ Defined as employed persons who usually work ≥35 hours per week at all jobs combined.

^§^ HHS *Region 1:* Connecticut, Maine, Massachusetts, New Hampshire, Rhode Island, and Vermont; *Region 2:* New Jersey, New York, and the territories Puerto Rico and the Virgin Islands; *Region 3:* Delaware, District of Columbia, Maryland, Pennsylvania, Virginia, and West Virginia; *Region 4:* Alabama, Florida, Georgia, Kentucky, Mississippi, North Carolina, South Carolina, and Tennessee; *Region 5:* Illinois, Indiana, Michigan, Minnesota, Ohio, and Wisconsin; *Region 6:* Arkansas, Louisiana, New Mexico, Oklahoma, and Texas; *Region 7:* Iowa, Kansas, Missouri, and Nebraska; *Region 8:* Colorado, Montana, North Dakota, South Dakota, Utah, and Wyoming; *Region 9:* Arizona, California, Hawaii, Nevada and the territories American Samoa, Commonwealth of the Northern Mariana Islands, Federated States of Micronesia, Guam, Marshall Islands, and Republic of Palau; *Region 10:* Alaska, Idaho, Oregon, and Washington.

^¶^ Significantly exceeded the epidemic threshold.

**Figure 1:**
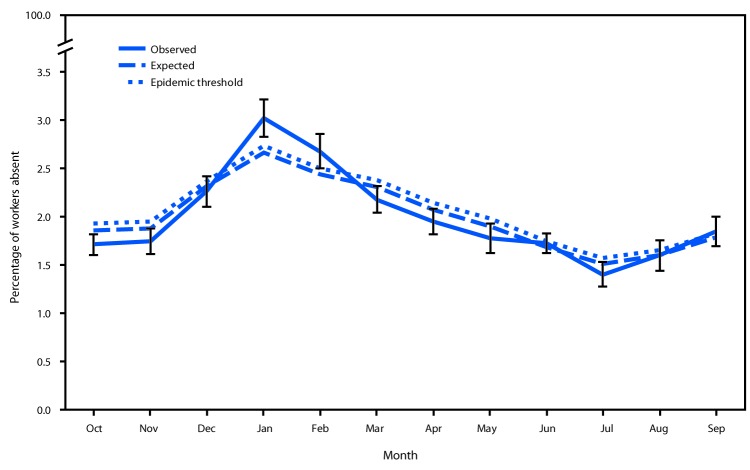
Observed* versus expected**^†^** health-related workplace absenteeism**^§^** among full-time workers**^¶^** — Current Population Survey, United States, 2017–18 influenza season * Error bars represent 95% confidence intervals (CIs) for point estimates. ^†^ Expected values based on monthly averages for the previous five seasons. Epidemic threshold is the upper 95% CI for expected values. ^§^ Defined as working <35 hours during the reference week because of illness, injury, or other medical issue. ^¶^ Defined as employed persons who usually work ≥35 hours per week at all jobs combined.

**Figure 2:**
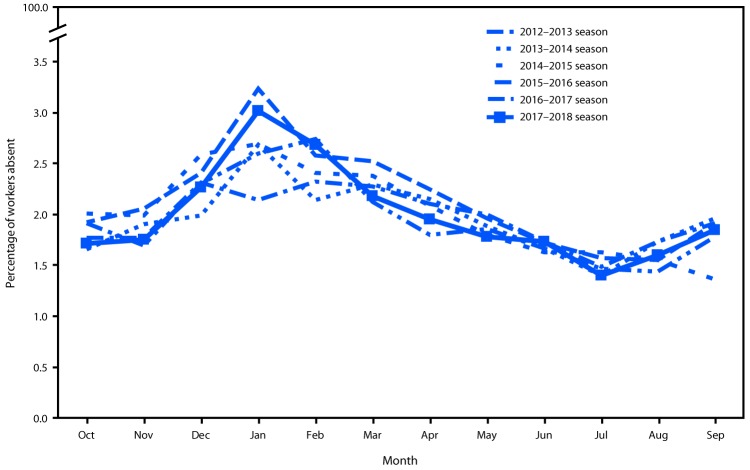
Health-related workplace absenteeism* among full-time workers**^†^** — Current Population Survey, United States, 2012–13 through 2017–18 influenza seasons * Defined as working <35 hours during the reference week because of illness, injury, or other medical issue. ^†^ Defined as employed persons who usually work ≥35 hours per week at all jobs combined.

The epidemic threshold was significantly exceeded for the following subgroups: male workers in January and February; workers aged 45–64 years in January and February; workers in HHS Region 6 in January and February and in Region 9 in December and March; and workers in management, business, and financial occupations and installation, maintenance, and repair occupations in January and in production and related occupations in February (Table) Regional absenteeism peaks corresponded to concurrent peaks in influenza-like illness (ILI) activity in those regions.^††^

## Discussion

These findings for 2017–18 are consistent with those of a study using conventional surveillance data, which characterized that season as a high severity influenza season that accelerated in November and peaked in late January and early February (*4*). For some time, it has been recognized that health-related workplace absenteeism correlates well with the prevalence of ILI and reaches seasonal peaks in conjunction with influenza activity as measured by other established methods during epidemics and pandemics (*7*). NIOSH’s experience with workplace absenteeism surveillance during the 2009–10 influenza A(H1N1) pandemic indicated that peak workplace absenteeism was correlated with the highest occurrence of both ILI and influenza-positive laboratory tests (*2*). For this reason, data on workplace absenteeism have been used as a nonspecific or syndromic indicator of the occurrence of ILI in the community in various settings (*2*). Typically, these data have been collected in near real-time from individual or small, nonprobability samples of sentinel worksites, often as part of ad hoc surveillance efforts associated with particular events or outbreaks and intended to serve as epidemic early warning systems. Although timely, such systems are typically difficult to sustain and provide data that are generally less stable and reliable, of lower quality, and subject to increased bias (*2*). Samples from such systems also tend to be small and nonrepresentative and, therefore, less able to reflect variation in patterns of absenteeism across geographic, demographic, and occupational subgroups (*2*).

NIOSH’s continuous population-based surveillance of absenteeism makes use of survey data that are valid, reliable, and nationally representative (*2*). Although the 1-month lag precludes CPS data from being sufficiently timely to be used as an early warning system, they are timely enough to provide a useful direct measure of a pandemic’s impact on the working population and an indirect measure of a pandemic’s economic impact (*8*). CPS data also provide information that can be used to maintain situational awareness during the interpandemic period, to evaluate the impact of control measures implemented during a pandemic (e.g., social distancing measures), and to inform future pandemic preparedness and response planning.

The associations of ILI and workplace absenteeism with occupation and other demographic characteristics are complex and mediated by factors such as vaccination coverage and access to paid sick leave (*9*). More study using additional data sources is needed to fully understand the reasons for increases in absenteeism related to sex, age, or specific occupations that are identified by these surveillance analyses.

The findings in this report are subject to at least five limitations. First, operationalized, health-related workplace absenteeism includes absences because of injuries, preventive care, and illnesses unrelated to influenza, which could attenuate or confound absenteeism’s relation to influenza activity; however, the correlation between absenteeism and influenza activity has repeatedly been found to be strong in the U.S. population. Second, the survey data used for these analyses were self-reported or reported by a family member proxy respondent. Although the 1-week CPS recall period is very short, in principle, these data are subject to recall, social desirability, and other biases that affect self- and proxy-reported data. Third, monthly absenteeism estimates are based on 1-week measures and could have underestimated or overestimated the actual prevalence for any given month in a way not reflected in the 95% CIs. Fourth, the nature of CPS data only allows for calculation of health-related absenteeism among full-time workers; patterns of absenteeism and its relation to ILI might be different among part-time workers. Finally, the amount of overlap between absenteeism and conventional measures of medically attended illness is unknown and variable. Thus, some uncertainty exists regarding the extent to which absenteeism adds to conventional measures of influenza morbidity.

Because workers often share office space and equipment and have frequent face-to-face contact, the workplace can be an important setting for influenza transmission. Nearly two thirds of adults in the United States participate in the workforce, and estimates of influenza attack rates for working-aged adults (18–64 years) can be as high as 14.3% in a given influenza season (*10*). Surveillance of workplace absenteeism can provide an important supplementary measure of a pandemic’s impact because conventional morbidity and mortality statistics might not fully reflect the disruption caused to the social and economic life of the community. Workplace absenteeism is also one component of the World Health Organization’s Pandemic Influenza Severity Assessment impact indicator.^§§^

Vaccination and nonpharmaceutical interventions recommended for everyday use, such as staying home when sick, covering coughs and sneezes, practicing hand hygiene, and routinely cleaning frequently touched surfaces, are the most effective ways to prevent influenza transmission during seasonal epidemics, both in the community and in the workplace (*5*). During a pandemic, additional personal and community nonpharmaceutical interventions might be recommended, including social distancing measures in workplaces (*5*). NIOSH makes current and past seasons’ absenteeism surveillance results available online (*6*). State and local health authorities, as well as employers, might wish to consult these results when developing and targeting prevention messages and use them to monitor long-term trends for their jurisdiction during interpandemic periods. Analysis of aggregated absenteeism data from multiple seasons might also help identify occupational groups at higher risk for influenza transmission.

**Summary**

**What is already known about this topic?**

Surveillance using mortality, health care encounters, and laboratory data does not reflect the full extent of influenza morbidity. CDC’s National Institute for Occupational Safety and Health conducts monthly monitoring of health-related workplace absenteeism.

**What is added by this report?**

During the 2017–18 influenza season, absenteeism increased sharply in November and peaked in January, at a level significantly higher than the average during the previous five seasons. Workers who were male, aged 45–64 years, and working in certain U.S. Census regions and occupations were more affected than were other subgroups.

**What are the implications for public health practice?**

Workplace absenteeism is an important supplementary measure of influenza’s impact on the working population that can inform prevention messaging and pandemic preparedness planning.

## Footnotes

* HHS Region 6 includes the states of Arkansas, Louisiana, New Mexico, Oklahoma, and Texas. https://www.hhs.gov/about/agencies/iea/regional-offices/index.html.

† HHS Region 9 includes the states of Arizona, California, Hawaii, and Nevada. https://www.hhs.gov/about/agencies/iea/regional-offices/index.html.

§ https://www.census.gov/programs-surveys/cps/technical-documentation/methodology/non-response-rates.html.

¶ HHS Regions are used for consistency with geographic regions used in CDC’s ILI surveillance. https://www.cdc.gov/flu/weekly/overview.htm.

** Occupational groups correspond to the CPS Major Occupational Group recodes, which are groupings of Census Occupation Codes (https://www2.census.gov/programs-surveys/cps/methodology/Occupation%20Codes.pdf). The Census Occupation Codes are, in turn, based on the 2010 Standard Occupational Classification codes promulgated by the Bureau of Labor Statistics (https://www.bls.gov/soc/2018/home.htm).

†† https://gis.cdc.gov/grasp/fluview/fluportaldashboard.html.

§§ http://www.who.int/influenza/surveillance_monitoring/pisa/pisaindicators/en/.
